# Supplementary material for: Predicting visual function by interpreting a neuronal wiring diagram
Source: Nature. 2024 Oct 2;634(8032):113–23. doi: 10.1038/s41586-024-07953-5 (PMC11446822; doi:10.1038/s41586-024-07953-5)
Supplement: Supplementary file 6 — CRF and ERF predictions for individual TmY4 and TmY9 cells. Analogous to Supplementary Data 3, but for TmY target types. Shown are the top four monosynaptic pathways, the strongest pathway passing through each of the top ten intermediary types (ranking from Extended Data Fig. 7), and the trisynaptic pathway Tm1–TmY–Dm3–TmY (see the section entitled Prediction of spatial normalization). [file 41586_2024_7953_MOESM6_ESM.zip › DataS4/TmY9q/720575940615708566.pdf]

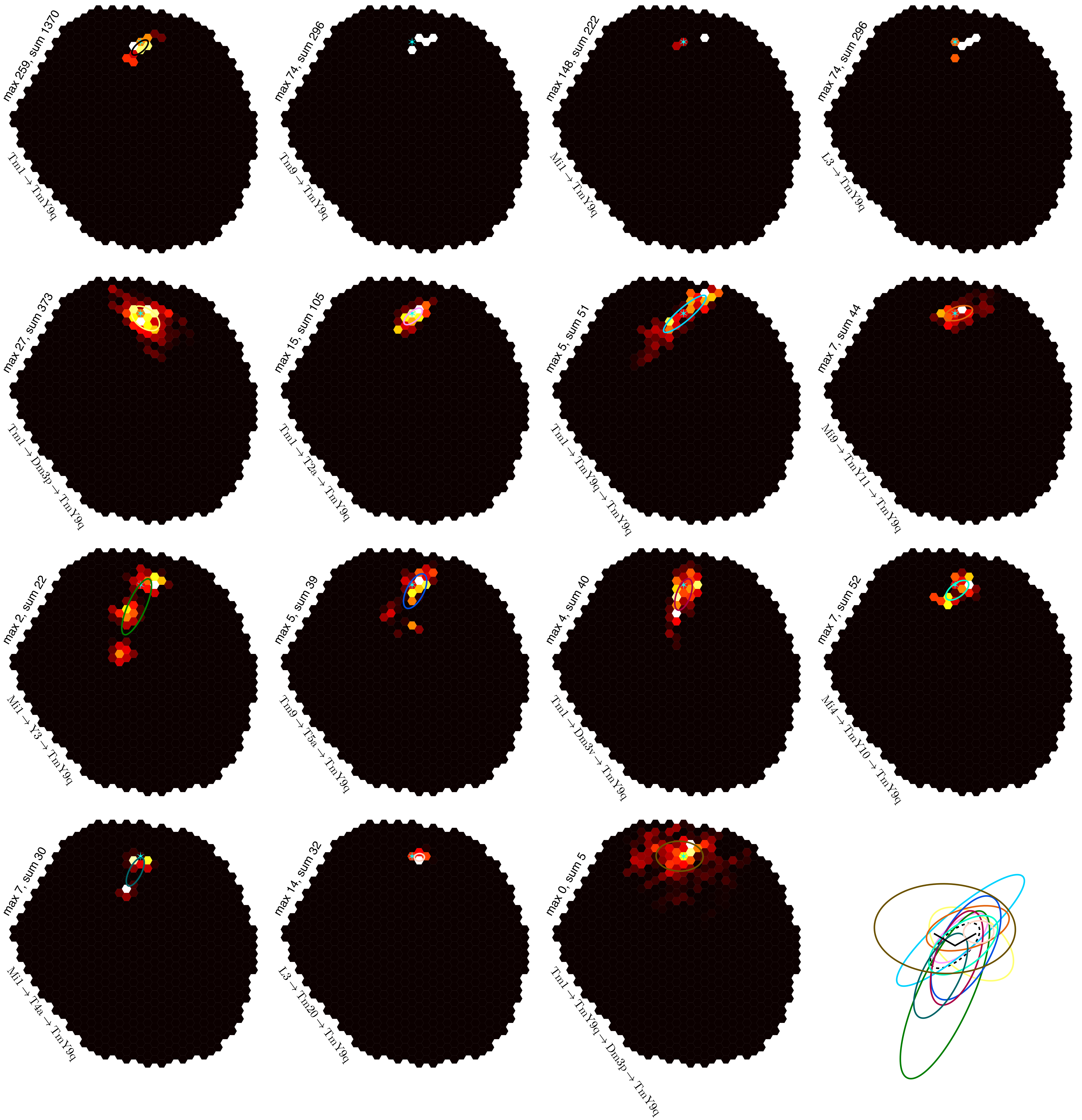
max 259, sum 1370
Tm1 → TmY9q
max 74, sum 296
Tm9 → TmY9q
max 148, sum 222
Mi1 → TmY9q
max 74, sum 296
L3 → TmY9q
max 27, sum 373
Tm1 → Dm3p → TmY9q
max 15, sum 105
Tm1 → T2a → TmY9q
max 5, sum 51
Tm1 → TmY9q → TmY9q
max 7, sum 44
Mi9 → TmY11 → TmY9q
max 2, sum 22
Mi1 → Y3 → TmY9q
max 5, sum 39
Tm9 → T5a → TmY9q
max 4, sum 40
Tm1 → Dm3v → TmY9q
max 7, sum 52
Mi4 → TmY10 → TmY9q
max 7, sum 30
Mi1 → T4a → TmY9q
max 14, sum 32
L3 → Tm20 → TmY9q
max 0, sum 5
Tm1 → TmY9q → Dm3p → TmY9q
